# Mobile Health Tobacco Cessation Interventions to Promote Health Equity: Current Perspectives

**DOI:** 10.3389/fdgth.2022.821049

**Published:** 2022-06-30

**Authors:** Samuel L. Battalio, Angela F. Pfammatter, Kiarri N. Kershaw, Alexis Hernandez, David E. Conroy, Bonnie Spring

**Affiliations:** ^1^Department of Preventive Medicine, Northwestern University Feinberg School of Medicine, Chicago, IL, United States; ^2^Department of Kinesiology, The Pennsylvania State University (PSU), University Park, PA, United States

**Keywords:** disparities (health, mHealth (mobile health), tobacco and tobacco product, cessation, disparities (health racial)

## Abstract

Although US tobacco use trends show overall improvement, social disadvantage continues to drive significant disparities. Traditional tobacco cessation interventions and public policy initiatives have failed to equitably benefit socially-disadvantaged populations. Advancements in mobile digital technologies have created new opportunities to develop resource-efficient mobile health (mHealth) interventions that, relative to traditional approaches, have greater reach while still maintaining comparable or greater efficacy. Their potential for affordability, scalability, and efficiency gives mHealth tobacco cessation interventions potential as tools to help redress tobacco use disparities. We discuss our perspectives on the state of the science surrounding mHealth tobacco cessation interventions for use by socially-disadvantaged populations. In doing so, we outline existing models of health disparities and social determinants of health (SDOH) and discuss potential ways that mHealth interventions might be optimized to offset or address the impact of social determinants of tobacco use. Because smokers from socially-disadvantaged backgrounds face multi-level barriers that can dynamically heighten the risks of tobacco use, we discuss cutting-edge mHealth interventions that adapt dynamically based on context. We also consider complications and pitfalls that could emerge when designing, evaluating, and implementing mHealth tobacco cessation interventions for socially-disadvantaged populations. Altogether, this perspective article provides a conceptual foundation for optimizing mHealth tobacco cessation interventions for the socially-disadvantaged populations in greatest need.

## Introduction

Efforts to reduce tobacco use in the US have failed to equitably benefit many socially-disadvantaged populations. Decreased tobacco use over the past two decades is largely because well-resourced, socially-advantaged populations have responded well to public policy efforts and tobacco cessation interventions, reaching endgame levels (<5%) of tobacco use (1, 2). Meanwhile, the prevalence of tobacco use among those who experience significant social disadvantage has either stopped declining or continues to worsen (2). Population-level statistics show significant disparities based on racial and ethnic minority status, poverty, education, and rurality (3–6). Even though quit attempts are common among socially-disadvantaged tobacco users, they rarely succeed, largely because of reliance on non-evidence-based cessation interventions (1, 7, 8). Scalable, effective tobacco cessation interventions with sufficient reach to permeate socially-disadvantaged populations are necessary to redress tobacco use disparities.

Increased recognition of access barriers has led many tobacco experts to turn to digital modalities as a delivery channel for equitable reach. Smartphones, in particular, have become ubiquitous, providing internet connectivity even for those who lack broadband connectivity (9, 10). The ubiquity of smartphones has prompted delivery of evidence-based tobacco cessation interventions to pivot toward mobile health (mHealth) tools. Relatedly, recent best practice guidance encourages state-run programs to leverage digital tools (both web and smartphone) to facilitate mass reach (11). Additionally, because of the barriers it created to in-person health care delivery, the COVID-19 pandemic further fueled the uptake of digitally supported interventions, either as standalone treatment or as a means of supporting remotely-delivered, connected counseling. Given that the pandemic simultaneously ushered in both an exacerbation of existing health disparities and increased use of technology-supported intervention (12–14), it is essential to examine whether the use of digital technologies played any role in worsening disparities.

In this paper, we provide our perspectives on the current state of the science surrounding mHealth interventions to promote health equity in cessation of tobacco use. We will review research on how social determinants of health (SDOH) contribute to tobacco use disparities. Against that backdrop, we propose a conceptual model that can guide the development of mHealth tobacco cessation interventions to address SDOH. In that manner, we hope to ensure that mHealth interventions for tobacco cessation serve the socially disadvantaged populations with greatest need.

## The Tobacco Use Disparities Landscape Seen Through a Lens of Social Determinants of Health

### Defining Social Determinants of Health

It is now accepted that health disparities, including those related to tobacco use, are driven by an interplay of factors that function within and across multiple levels of influence. These factors are commonly referred to as social determinants of health (SDOH). Although widely used, the term SDOH has different meanings based on one's field and scientific background. The term SDOH was originally used as a “catch-all” to designate all health influences that originate and function outside of explicit “medical care.” Even though this usage persists in some sectors today, SDOH are now conceptualized with greater nuance (15, 16).

Most current definitions share two unifying themes. First, modern definitions tend to place greater emphasis on extra-individual rather than intra-individual influences. Second, SDOH are now conceptualized less as a collection of independent influences, and more as factors that function interdependently to influence health across various domains (e.g., biological, behavioral, physical/built environment) and levels (e.g., individual, interpersonal, community) (17).

## Social Determinants of Tobacco Use

A substantial body of research links various SDOH with tobacco use through direct and indirect mechanisms. Below, we provide a brief, non-exhaustive overview of particularly well-studied SDOH and the mechanisms by which they are thought to influence tobacco use.

### Socioeconomic Status

The most substantially researched SDOH are probably those that contribute to individual-level socioeconomic status (SES). The primary SES factors that have been studied include education, income, occupational status, and insurance status (18). Findings indicate that individuals living in poverty smoke for twice as many years as those above the poverty line (19). Moreover, even though the number of quit attempts between those living above vs. below the poverty line are comparable, success at quitting is substantially lower among those of low SES (19, 20).

Increasingly, it is recognized that individual SES may be a proxy for extra-individual influences on tobacco use. Businelle et al. (18) modeled the direct and indirect relationships between individual SES and smoking cessation status. They found that individual SES, measured by combining education, income, insurance status, and employment status, had both direct and indirect effects on smoking cessation status. Indirect effects were mediated partly by extra-individual factors (self-reported neighborhood disadvantage and social support), and partly by intra-individual factors (negative affect and personal agency). To understand extra-individual factors that may share variance with SES, Cambron et al. (21) leveraged ecological momentary assessment to model the relationship between individual SES, social contextual factors (e.g., pro-smoking social contexts, cigarette availability), and smoking lapse. Their finding—that low-SES individuals were more likely to be exposed to pro-smoking social contexts—highlights the fact that individual-level measures of SES may actually encompass variance that is better explained by extra-individual SDOH effects.

### Neighborhood Built Environment

Neighborhood environmental and contextual factors have also been shown to play a large role in tobacco use *via* numerous mechanisms. Upstream neighborhood-level factors in low-SES neighborhoods include greater density of tobacco marketing (22) and tobacco retail outlets (23), which have been directly linked with tobacco use. Ecological momentary assessment and geospatial information systems (GIS) methods show that exposure to point-of-sale tobacco is associated with greater likelihood of same day lapse (24). Moreover, numerous measures of neighborhood-level social disadvantage, including area-level unemployment, low safety, high crime, and low social cohesion, are associated with greater rates of tobacco use and lower rates of tobacco cessation success (25–27). Importantly, these neighborhood-level associations with tobacco use typically remain significant even after controlling for individual-level characteristics (28–31). Although the pathways by which neighborhood social disadvantage affect tobacco use remain unclear, some proposed mechanisms include reduced internal locus of control and elevations in stress and negative affect, each of which has been shown to be an independent driver of tobacco use (32).

### Rurality

Living in a rural area has been established as a longstanding, independent predictor of tobacco use. Longitudinal analyses from the past few decades show that prevalence of tobacco use has declined more rapidly in urban than rural settings (33, 34). Recent estimates suggest that individuals living in rural settings are almost twice as likely as those living in urban areas to smoke cigarettes. Rural residents also have 20% higher lung cancer incidence (35).

Longitudinal analyses suggest that the mechanisms by which rurality influences tobacco use have changed over time. Doogan et al. (34) found that the covariation between rurality and tobacco use during 2007 was statistically explained by differences between urban and rural populations in terms of sociodemographic and psychosocial risk factors (e.g., age, race, education, income, employment status, anxiety, depression, health insurance status). However, in 2014, rurality was directly associated with tobacco use, over and above the effect of those covariates. They posit that this shift may be explained, in part, by differential reach of tobacco control policy efforts, which have preferentially targeted densely-populated, non-rural areas.

Despite being one of the more widely recognized and studied social determinants of tobacco use, problems with operationalizing rurality have posed questions, produced inconsistent findings, and sparked debate. Numerous operational definitions exist, and, as discussed in-depth elsewhere, each carries potential advantages and disadvantages (36–38).

### Racial and Ethnic Background

Factors associated with racial and ethnic background play a significant role in tobacco use and its resulting health consequences. Although not a SDOH, racial and ethnic background is partly a proxy for a variety of the SDOH (e.g., racism, discrimination). Race and ethnicity are also, in and of themselves, independent reflections of all the numerous potential exposures that one's identity encapsulates. Estimates consistently show that cigarette use is most prevalent among American Indian/Alaska Natives (23%), followed by African Americans/Blacks (15%) and Non-Hispanic Whites (15%), Hispanic/Latinx (10%), and non-Hispanic Asians (7%) (39). However, an overarching view of tobacco use trends only tells part of the story. For example, despite having comparable tobacco use rates, those who are non-Hispanic Black are more likely than those who are non-Hispanic White to attempt to quit, and those attempts are less likely to be successful (40).

Multiple SDOH contribute to the association between tobacco use and racial and ethnic background. Experiences of discrimination are associated with greater dependence on tobacco products among multiple racial and ethnic minority populations (41). Additionally, SES-related SDOH tend to explain part of the relationship between racial and ethnic background and tobacco use, but the relationship varies depending on racial and ethnic background. For example, among Mexican Americans, some data indicate that financial strain and insurance status are more influential indicators of capacity to quit smoking than are other SES indicators, such as education and income (42). Similarly, among African Americans, unemployment, at both the individual and neighborhood levels, appears to be a particularly important indicator of smoking cessation capacity (26). Upstream factors to which racial and ethnic minority groups tend to be disproportionally exposed include a higher volume of targeted tobacco advertisements, (43, 44) greater density of tobacco retailers, (45) and weak implementation of tobacco control policies (46, 47).

## mHealth Tobacco Use Interventions For Socially-Disadvantaged Populations

Although research in mHealth tobacco cessation intervention has grown exponentially, this body of evidence is still in its infancy. A recent systematic review of mHealth tobacco cessation interventions identified 18 trials, of which the majority were identified as pilot or feasibility trials (48). Although several studies had acceptable representation of populations that exhibit tobacco use disparities, few explicitly developed an intervention to redress a disparity or to address SDOH. That said, existing work can provide valuable insights to support developing mHealth tobacco cessation interventions that explicitly advance health equity. So far, this intervention development research has tended to take one of two broad approaches.

In one approach, which we term the *SDOH Targeting Approach*, researchers select a specific, underrepresented group (usually in a specific socio-geographic context). Then, they leverage models of SDOH to create a customized mHealth intervention package designed to engage individuals who match the demographic and socioenvironmental context of interest. For example, a researcher may take an existing mHealth intervention designed for the general population, and use a SDOH framework [e.g., Cultural Accommodation of Substance Abuse Treatment framework (49)] to adapt the treatment linguistically and culturally for adult Hispanic/Latinx cigarette smokers recruited from an outpatient community health clinic in a rural town. The strength of this approach is that the resulting intervention package is likely to be acceptable and feasible because it is adapted to the target population. The intervention is also likely to retain its efficacy so long as the adaptation has not undone any core components of the validated intervention package.

The other common approach, which we refer to as the *Generalist Approach*, creates or deploys an intervention designed to meet the broad needs of the overall population of tobacco users. Researchers then pilot test the intervention among socioeconomically and socioculturally diverse samples to examine the intervention's acceptability and feasibility in socially-disadvantaged populations. Assuming that no explicit SDOH-based adaptation is necessary, this approach may result in an intervention package that is feasible, acceptable, and generalizable across a broad variety of subpopulations and contexts.

Along with benefits, each of these approaches has potential for significant drawbacks. For example, the SDOH Targeting Approach commits significant research dollars and resources to develop an intervention that is intentionally designed for a niche context, limiting potential generalizability and reach. Although this degree of specific customization might be necessary, that assumption should be rigorously and empirically tested. By contrast, without adhering to an SDOH framework, a Generalist Approach risks alienating subgroups or underperforming in high-need contexts. The shared risk is that either approach may develop a feasible and acceptable pilot intervention, and then move pre-maturely to evaluate the treatment package in a full-scale randomized controlled trial (RCT). That progression leaves many important questions unanswered about the impact and cost of the intervention's components, their ability to accommodate and address SDOH, and the scalability of the full intervention package.

**Figure 1:**
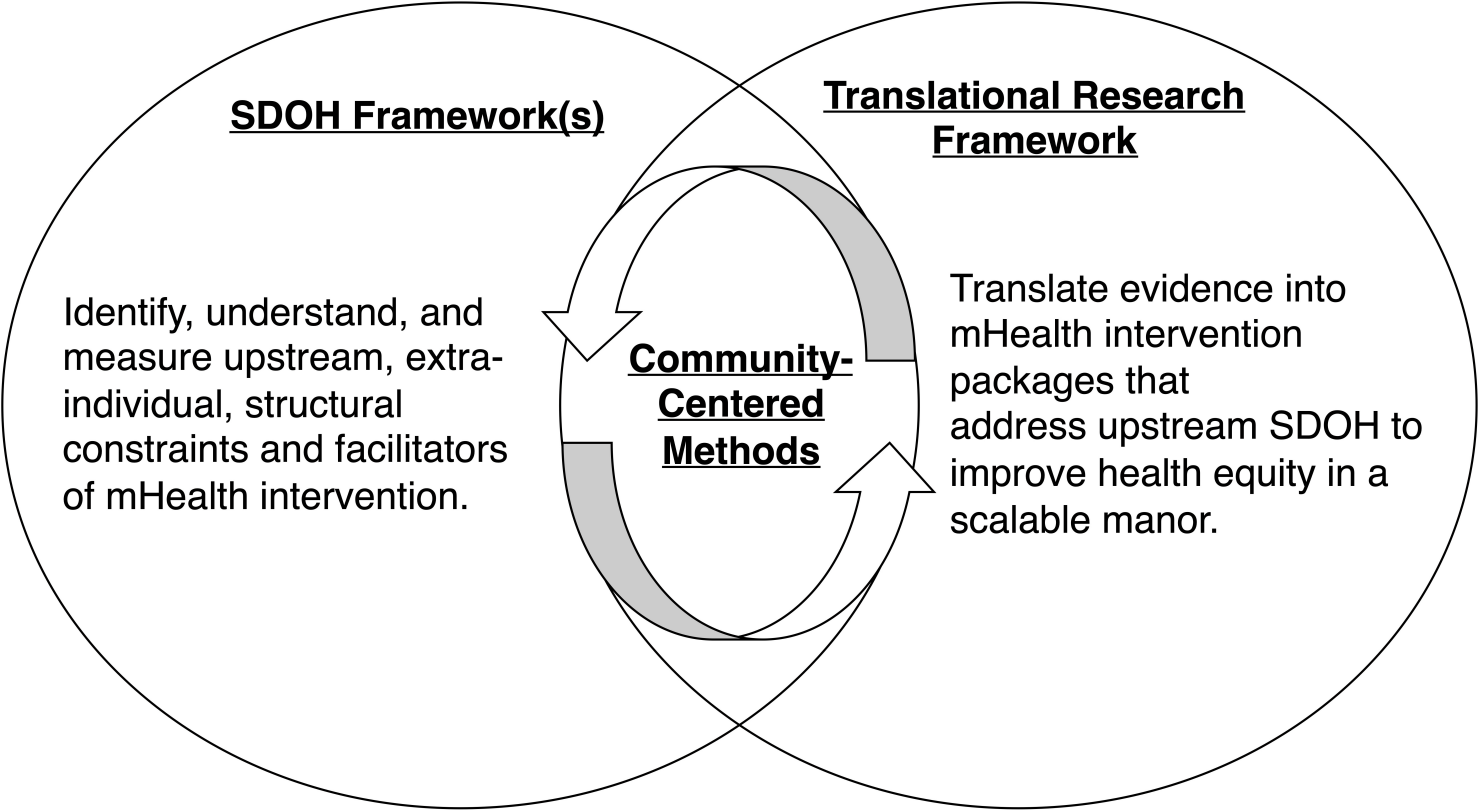
Conceptual model for equitable, SDOH-informed intervention development.

We propose that these risks can be minimized by creating an intervention development pipeline that integrates community-centered methods, SDOH framework(s), and a translational research framework. This conceptual model is depicted in Figure 1. SDOH frameworks are used to measure multilevel determinants and conceptualize their potential role in intervention. Some examples include the NIMHD Research Framework, (17) the social ecological model, (50) or the Healthy People 2030 model of SDOH (51). The translational framework ensures that researchers iteratively and systematically translate information gathered from SDOH frameworks into equitable intervention packages that contain components that improve health equity and forgo those that do not. Examples of translational research frameworks include the Multiphase Optimization Strategy (MOST), (52). Obesity-Related Behavioral Intervention Trials (ORBIT) model, (53) the experimental medicine approach, (54) or the Medical Research Council (MRC) framework (55). Importantly, we propose that community-engaged methods, such as those used within a Community-Based Participatory Research (CBPR) approach (56) or Citizen Science, (57) are necessary to integrate SDOH frameworks and the translational research framework, grounding each stage of intervention development with voices representing the perspectives of populations of need.

## Just-in-Time-Adaptive-Intervention (JITAI)

Perhaps one of the most novel and conceptually promising opportunities to promote health equity enabled by mHealth intervention technologies is the potential for real-time intervention that adapts dynamically to context (e.g., neighborhood stress exposures). Such interventions, termed just-in-time-adaptive-intervention (JITAI), leverage intensive longitudinal data collected by smartphones and wearable sensors to characterize an individual's real-time context, and then deliver an intervention in moments of elevated need or receptivity (58). Recent optimization research has begun to develop JITAI's for tobacco cessation.

In one study, we randomly delivered a digital stress management intervention prompt during minutes when a recently quit smoker was stressed vs. not stressed. The resulting JITAI will be the decision rule that specifies the optimal temporal context (stressed or not stressed) in which the digital intervention should be delivered to maximize protection against smoking relapse (59). We believe that such JITAIs have promise to promote health equity because they can deliver interventions that act downstream on individual-level determinants (e.g., stress management behaviors) to trigger them when needed to address upstream environmental and contextual SDOH (e.g., environmental stress triggers, such as real-time exposure to tobacco retailers or neighborhood physical danger).

### Application of the Proposed Conceptual Model

**Figure 2:**
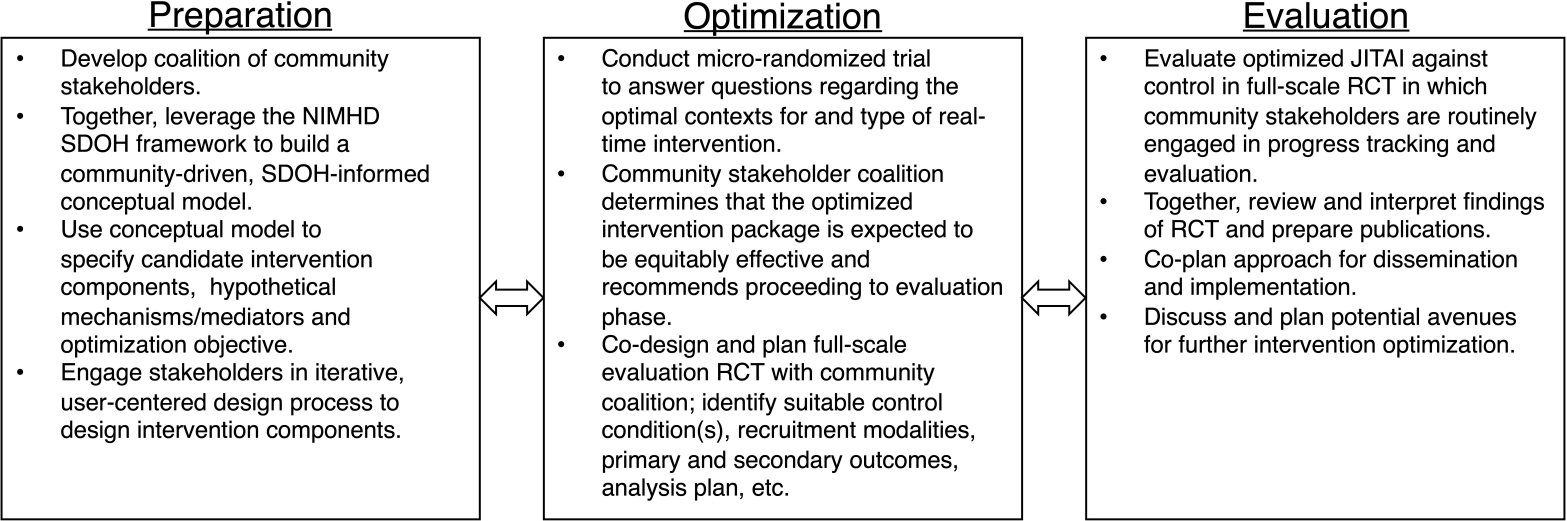
Hypothetical application of proposed conceptual model using the multiphase optimization strategy (MOST).

To illustrate, we apply our proposed conceptual model to this line of intervention development research aimed at developing a stress management JITAI to protect against smoking relapse. In Figure 2, we propose a non-exhaustive, hypothetical application of our conceptual model using MOST as our guiding translational research framework. MOST integrates insights from engineering, statistics, and behavioral intervention science, for the “development, optimization, and evaluation of behavioral, biobehavioral, and biomedical interventions” (52). Numerous, comprehensive explanations of MOST methodology exist elsewhere [e.g., (60)].

Multiphase Optimization Strategy is particularly well-equipped to handle the complexity of SDOH and to build interventions that are both effective and scalable enough to tangibly redress tobacco disparities. MOST requires systematic preparation phase work that culminates in measurable objectives and a sound conceptual model to guide subsequent optimization and evaluation. Laying a sound research foundation that links intervention components to SDOH is particularly valuable for developing interventions to promote health equity and eventual uptake. MOST is also specifically used for building interventions that are multicomponent to target different determinants of a risk behavior. To address specific SDOH, researchers may need to systematically add new components, as well as remove or restructure existing components, tasks for which MOST is particularly well-suited.

In our hypothetical intervention development example depicted in Figure 2, we highlight specific activities that use community-based participatory research to infuse consideration of SDOH into preparation, optimization, and evaluation of the intervention. For example, during the preparation phase, researchers form a coalition of community stakeholders and together leverage the NIMHD SDOH framework to build an SDOH-informed conceptual model for optimizing the stress management JITAI to address upstream SDOH. From the foundation of this conceptual model, researchers and community stakeholders identify candidate components (e.g., real-time stress management Apps, triggered based on proximity to high crime zones and tobacco retailers), hypothetical mechanisms and the optimization objective. Researchers then engage community stakeholders in user-centered design of the intervention, ensuring the intervention is appropriate for the intended population. Next, during optimization, researchers conduct a micro-randomized trial (MRT) to answer questions regarding the optimal context(s) and form of real-time intervention. Based on findings from the MRT, the community coalition determines whether the optimized intervention package is expected to be equitably effective and, if so, recommends proceeding to evaluation. Researchers and the community coalition then co-design a full-scale evaluation RCT (e.g., identify suitable comparator condition(s), plan logistics such as recruitment and analytic plan). Finally, in the evaluation stage, the team evaluates the optimized JITAI using the co-designed RCT. Together, the team also reviews and interprets findings, prepares publications, and co-designs the approach to dissemination and implementation. Simultaneously, based on the continual optimization principle, the team discusses avenues to continue improving the intervention.

## Discussion

In this article, we provided a current perspective on the science surrounding mHealth interventions to promote equitable intervention for tobacco cessation. We provided a non-exhaustive overview of how SDOH contribute to tobacco use disparities. Against that backdrop, we reviewed current mHealth tobacco cessation intervention research, highlighting that much of this work is in the early intervention development phase (e.g., formative design, pilot trials), and pointing out important considerations to ensure that intervention development pipeline outputs interventions that tangibly work to redress tobacco use disparities. We propose a conceptual model for developing interventions that uses community-centered methods to systematically integrate SDOH framework(s) with a suitable translational research framework. We close by suggesting several benefits of using MOST as a translational research framework, including capacity to integrate SDOH framework(s) and community-centered methods, and adept handling of multicomponent interventions. We also elevate the JITAI for its potential to support intervention that dynamically accommodates upstream, contextual SDOH exposures.

Despite burgeoning research support for mHealth in tobacco cessation, from the lens of health equity, many limitations may yet be uncovered. Given the relative novelty of mHealth, we may not necessarily know at this stage whether mHealth creates additional barriers or complications to intervening on traditionally marginalized tobacco users. Although mHealth technologies have become increasingly ubiquitous, questions remain regarding their acceptability and feasibility across society's most socially-disadvantaged populations. Indeed, numerous barriers may disproportionately impact socially-disadvantaged populations, including limited technology and internet accessibility, low digital literacy, and linguistic barriers (61, 62). For these reasons, as we continue to uncover opportunities and challenges posed by implementing mHealth for tobacco cessation, it is essential that researchers approach intervention development systematically, in partnership with the community, and through the lens of SDOH.

## Data Availability Statement

The original contributions presented in the study are included in the article/supplementary material, further inquiries can be directed to the corresponding author/s.

## Author Contributions

SB led the conceptualization, drafting, and editing of this manuscript. AP, KK, AH, DC, and BS assisted with conceptualization, drafting, and editing. All authors contributed to the article and approved the submitted version.

## Conflict of Interest

The authors declare that the research was conducted in the absence of any commercial or financial relationships that could be construed as a potential conflict of interest.

## Publisher's Note

All claims expressed in this article are solely those of the authors and do not necessarily represent those of their affiliated organizations, or those of the publisher, the editors and the reviewers. Any product that may be evaluated in this article, or claim that may be made by its manufacturer, is not guaranteed or endorsed by the publisher.
